
JOURNAL INFORMATION
==============================
NLM Title Abbreviation: Med Chir Rev
NLM Journal ID: 0034617

The Medico-Chirurgical Review

Publisher: Biomedical Journal Digitization Project

ARTICLE INFORMATION
==============================
PMCID: PMC4966268
PMID: 29917638
Article version: 1
Subjects: Articles

Hospital Practice

Print publication date: 1828 Oct 1
Volume: 9
Issue: 18
First page: 529
Last page: 552
1828 Sep
License: This work is free of known copyright restrictions. For more information, please see PMC Back Issue Digitization.
License URL: https://creativecommons.org/publicdomain/mark/1.0/

==============================

63. On Visceral Neuralgia. By M. Jolly. (Part the Second.)

In this second paper M. Jolly pursues the investigation of a very difficult, but a very important subject. Our author quotes a passage from Bichat, which shews that that illustrious pathologist did not overlook this important class of human afflictions.

"There are," says Bichat, "colicks essentially nervous, and which are totally independent of any local affection of the serous, mucous, or muscular coats of the intestines. These colicks have their seat evidently in the nerves emanating from the semilunar ganglia, and spread along the whole arterial system of the abdomen. "*They are veritable neuralgiæ of the nervous system of the organic life,* although these neuralgiæ have nothing in common with tic douloureux, &c."

In this last expression Bichat seems to have forgotten that the visceral nerves do not feel in the same manner as the cerebro-spinal nerves, and this difference of sensibility is quite sufficient to account for the greater quantity of pain that attends tic douloureux when affecting the former class of nerves. The affection seems really to be of the same nature in both cases, and only modified by the characters of the two classes of nerves. But, as we shall give a greater extension to this subject, when reviewing that part of Dr. Macculloch's work that treats of Neuralgia, we shall here confine ourselves to the cases brought forward by M. Jolly. The following passage, however, is deserving of consideration, en passant.

"In the neuralgiæ of the trisplanchnic, as in those of the cerebro-spinal nerves, the *pain* is not the only sensible phenomenon. Generally there is an afflux of blood to the tract of the nerves affected, as well as to the adjacent tissues; and as these two phenomena constitute two of the essential characters of inflammation, they may help to account for that febrile reaction which takes place in so many of the intermittent neuralgiæ—and may go far to identify the neuralgiæ apyrecticæ with the neuralgiæ febriles."

Every day's experience of the last two years in this country will tend to confirm the above observation. The cause, whatever it may be, or whatever we may call it—marsh miasma, febrific miasma, dolorific miasma—produces in one individual a periodical head-ache—in another, an ague—in a third, a hepatalgia, too often mistaken for hepatitis—in a fourth, a gastralgia, treated by leeches, bleeding, blisters, &c. as gastritis. But more of this in another place.

*Case 1. Febrile neuralgia affecting the trisplanchnic nerves under the tertian form, and cured by antiperiodics after an unsuccessful trial of the depletory means.*

Mamade D———, aged 30 years, of sanguineous temperament, experienced, after an accouchement, a series of symptoms, apparently of an inflammatory character, and affecting the stomach, liver, kidneys, and uterus. Each attack, in short, resembled gastritis, hepatitis, nephritis, hysteritis, &c. according to the organ invaded, and each attack gave way to the usual depletory and antiphlogistic measures. But the intervals of relief were short and imperfect—and a few days generally sufficed to bring the malady back in some new place. At length, after the patient was reduced to a state of great debility, it was observed that the disorder made its appearance regularly every third day, whatever was the organ selected for its seat. Having arrived at the lowest ebb of weakness, and when depletive measures could no longer be thought of, the patient continued to be harrassed with most distressing pains in almost all the viscera supplied by the trisplanchnic nerves. These pains were unequivocally periodical, returning every other day at a fixed hour, generally about eleven o'clock in the forenoon, commencing with a chilliness, head-ache, and vertigo, then affecting successively the epigastrium, the right hypochondrium, and the right side of the hypogastrium. The pains were accompanied by violent vomiting, and by such painful distension of the abdomen, especially about the region of the liver, that she could scarcely bear the weight of the bed-clothes. This state lasted from twelve to fifteen hours, terminating in a gentle warm perspiration, and being succeeded by an interval of nearly perfect health. In these intervals, there was neither pain nor even tenderness on pressure, in any part of the abdomen—no feverishness—no redness, but a slight whiteness of the tongue. In consultation with Dr. Chabanneau, it was agreed that the disease was a periodical neuralgia of the ganglionic system, and she was directed to take sixteen grains of the sulphate of quinine, with some opium and ether in the next interval. There was no return of the paroxysms till the eleventh day, when the patient thought proper to take a cathartic medicine, which immediately produced a recurrence of the attack. The quinine again stopped them permanently.

*Case* 2. *Quotidian Intermittent Neuralgia of the Uterus. By Dr. Duparque.* Madame R. C. aged 28 years, tall and robust, was safely delivered in the beginning of October, 1827. She did not nurse her child, and the catamenia were re-established in six weeks, continuing regular till the month of February, 1828, when they stopped, without any ostensible cause, but again returned, eight days after the expected period, accompanied by extraordinary pains chiefly affecting the right iliac region, shooting into the pelvis, and extending towards the opposite iliac region. These pains were accompanied by a bearing down, resembling that felt in labour. They were acute, lancinating, recurring every three or four minutes, and forcing the patient to cry out from their violence. They soon induced a degree of delirium and even convulsions. These attacks came on about mid-day, and lasted with more or less intensity till the evening, ceasing about midnight, and then permitting the exhausted patient to get sleep. In the mornings she appeared in good health, and free from pain; but at noon, the enemy regularly made his attack, as above-described. The catamenia continued to flow, and on the eighth day an accoucheur was called, under the idea that the patient was suffering from abortion. This idea, however, was given up, and it was decided that the disease was phlegmasia of the uterus. Venesection, leeches, repose, fomentations, lavements, diluents, and the usual remedies, were prescribed, but instead of relieving, they greatly aggravated the paroxysms. Dr. Duparque was called in on the 14th of March, the twenty-fifth day of the disease. The patient was now reduced to a complete state of emaciation—the appetite being nearly natural, the tongue moist and clean, the skin a little hot, the pulse quick and irregular, the abdomen soft and insensible to pressure, but this pressure excited some pain in the iliac and hypogastric regions. Nothing particular could be detected in either of these regions by manual examination. The os uteri felt rather turgid, and more open than natural, but not more so than during the catamenial period. The uterus itself did not appear to be at all enlarged. Dr. Duparque considered the disease as a regular PERIODICAL HYSTERALGIA, and promised the desponding patient a speedy cure. Eight grains of the sulphate of quinine were ordered to be taken during the next remission. The paroxysm did not return at noon, but only a few mitigated pains in the evening. There were no more attacks, but the patient continued the quinine for some days, by way of precaution.

*Case* 3. This case was recorded in the Journal of Vandermonde, so long ago as the year 1779, and bears upon the present investigation.

A female, aged 35 years, of melancholic temperament, was seized, after some domestic troubles, with *hysterical paroxysms*, evinced by great commotion in the bowels, globus hystericus, and a sense of insupportable anxiety. These paroxysms returned in the evening, lasted through the night, preventing sleep, and disappeared in the morning. She had been three months in this state when the narrator was consulted. The attacks, at first, were separated by intervals of a few days; but latterly that had been almost daily. She was cured, and very promptly too, by the sulphate of zinc.

*Case* 4. This is a case of intermittent rheumatism which happened in the person of Mr. Rumsey, a surgeon, and is recorded in the Edinburgh Medical and Surgical Journal for July, 1818.

"Fever came on in the afternoon, with a violent pain of the abdomen, a great sense of distention, and actual enlargement, with great flatulence. My own sensation was, that nothing would give relief but evacuations. An enema was given with little or no good effect, yet, in the course of a few hours I fell asleep, and awoke in the morning almost well, not expecting any renewal of disorder. But on the next evening I found myself suffering again precisely in the same manner. Obtained little or no relief from the injection, which I repeated, feeling as if nothing would relieve my pain but evacuations. Again I became well in the night, and was in the morning without fever or complaint. In the ensuing afternoon, at the usual hour, I was attacked, for the third time, with fever, my bowels were inflated, hard and full, and aching in the most distressing way. I began to suspect that the complaint was intermitting rheumatism, especially as I had many times had the most painful intermitting face-achs, which always gave way to bark. I had recourse to this remedy in substance, and, to my great satisfaction, escaped the paroxysm on the following day. My speedy recovery convinced me that the attack was intermitting rheumatism, and not inflammatory, as might, with reason have been suspected.

"I thought it a remarkable fact, not aware that rheumatism affected the muscles of the abdomen in this way, and still more remarkable, that, by their vicinity to the bowels, without any intelligible or direct communication, the viscera should be also affected, as the flatus and distention proved."

We admit that rheumatism is very frequently an intermittent disease; but we see no reason for concluding with Mr. Rumsey, that his was a case of that kind. Well might he wonder how rheumatism of the abdominal muscles should affect the abdomen with flatus and distention. The case is a very well marked one of the trisplanchnic neuralgia now under investigation. Mr. Rumsey states some particulars of another case, where a lady, after travelling in damp ground, became affected with an illness which, at first, was obscure, but afterwards assumed the type of a quotidian ague, "the paroxysms being accompanied by a distressing pain in the abdomen." This patient was quickly cured by bark. The following case shews clearly, we think, the connexion between neuralgia of the cerebro-spinal nerves, and that of the ganglionic nerves. It is also recorded by Mr. Rumsey.

*Case* 5. "Mrs. W——r, about 30 years old, had a sore throat, with some inconsiderable sloughs in it. After some days, without any remarkable occurrence, it got well, and, within a day or two, she was much troubled with face and tooth-ach. The pain was very acute, intermitting and returning once in the day, by a sudden accession. The use of the bark was begun, but producing a violent sickness and fainting, was discontinued. With warmth, and the use of wine, she soon lost the pain, and, except that she was weak, had for a few days no disease. Not many days had elapsed, however, before she complained of pain in the lumbar region, leading round the abdomen, and producing actual enlargement, as well as a distressing sense of fulness and flatus. It continued two or three hours, and then left her easy. The night and following day were free from pain, until 5 o'clock in the evening, at which hour it returned severely, affecting the intestines by distention, producing restlessness, irritability, and feverish quickness of pulse. I witnessed the same daily paroxysm, followed by its interval for five or six days, with the variation of observing a later hour, and being weaker in its attack."

The following case is recorded by M. Hutin.

*Case* 6. A female, aged 34 years, previously in good health, was awakened in the middle of the night with violent palpitation, accompanied by severe pain in the region of the heart, so that she was threatened with instant suffocation. In this distressing condition she remained till the morning, when all the symptoms suddenly vanished. At the same hour, in the succeeding night, the attack returned with equal violence—lasted till morning—and disappeared as before. The complaint now changed its form. The patient experienced, at irregular periods, violent pain in the stomach, accompanied by vomiting—or severe colicky pains in the bowels, region of the liver, or kidneys, which observed a periodical character. Repeated applications of leeches to the anus and warm baths were employed without success. Tonics and antispasmodics effected a cure.

That these visceral neuralgiæ assume the form of malignant intermittent fevers, our author is convinced, and the following case related by Torti, among others, is quoted in support of this opinion.

*Case* 7. A female became affected with a simple ague, accompanied by pains in the abdomen, vomiting and purging. It was in the third paroxysm when Torti was called to her, and no information was given to him respecting the periodicity of the complaint. The local symptoms were alone complained of. He found the patient cold, without pulse, the face deadly pallid, the eyes sunk, the nose pointed, the temples hollow. Torti thought her in articulo mortis; but re-action succeeded, and a remission followed. Next day the violent paroxysm of pain, sickness, and purging returned, and went through a certain period. Torti now perceived the nature of the complaint—prescribed the bark in large doses, and soon put an end to the paroxysms. We shall conclude our list of examples with the following case from M. BAILLY DE BLOIS.

*Case* 8. An Irish gentleman, aged 22 years, residing at Rome, was seized, in the month of August, 1822, with a paroxysm of ague, accompanied by excruciating pain in the abdomen. When Dr. Bailly arrived, he found the patient in a state of indescribable agitation. He was rolling about in his bed, pressing the abdomen with his hands, and uttering the most doleful lamentations. The tongue was rather white, without any redness at the point—no thirst—pulse full, strong, and developed. A pint of blood was taken from the arm, and 20 leeches were applied to the abdomen. In the evening, all the symptoms were dispersed, and the night was passed in tranquillity as was the following day. On the third day, however, he had a rigor in the morning, followed by fever, and the same excruciating pains in the abdomen which were described above. Twenty more leeches were applied to the abdomen, and a pound of blood abstracted from the arm. The paroxysm ceased in the evening—the night and succeeding day being free from complaint. On the fifth day, the symptoms returned with even increased violence, accompanied by great depression of spirits. The eyes of the physician now appear to have been opened. The paroxysm was allowed to proceed, and as soon as the apyrexia and cessation of pain took place, the sulphate of quinine was prescribed, and the attacks were stopped.

It is needless to accumulate more facts from various practical writers, to prove that examples of visceral neuralgia may be found in nervous colics, cholera morbus, periodical rheumatism, colica pictonum, &c. and that this disease, in its various shapes and domiciles, is still allied to, if not identified with—INTERMITTENT FEVER, not only as to cause, but as to treatment.

ANATOMICAL CHARACTERS. There are but few facts on record fit to demonstrate the nature of those lesions which constitute the trisplanchnic neuralgia. The difficulty of tracing the branches of these nerves, and still more of recognizing their physical lesions, renders this part of the investigation almost hopeless in the present state of neurological pathology. Lobstein, who has taken pains with the anatomy of the visceral nerves, especially the trisplanchic, found, on several occasions, unequivocal traces of inflammation in their structure, in the bodies of those who had complained of abdominal pains during life.

"On opening the body of a pregnant female, who had died of severe attacks of periodical vomiting, by which she had been harrassed for some months, accompanied by severe pain along the spine, Dr. Lobstein was unable to detect any trace of inflammation in the brain, spinal marrow, or any of the thoracic, abdominal, or pelvic viscera. Having removed the organs of the abdomen, he found the semilunar ganglion of an intensely red colour, and evidently inflamed. The trisplanchnic nerve was much larger than usual throughout its whole extent."

The same accurate observer found, in a young girl of six years of age, who had been long alternately affected with obstinate cough and spasmodic vomitings, the left division of the solar plexus highly inflamed, whilst the right was perfectly healthy. But it must be confessed that we have much, or rather every thing to learn respecting the morbid anatomy of the ganglionic nerves. The facts which we have brought forward in these two papers of M. Jolly, and the facts and reasonings which we shall shortly lay before our readers in the review of Dr. Macculloch's work, will, we hope, convince them that neuralgia is not confined to the nerves of relation, but extend also to the ganglionic system. The subject is highly deserving of attention, and its investigation will amply repay the labour expended on it. The periodical character of these visceral pains is too generally overlooked—the nature of the complaint is mistaken—the treatment is ineffectual, and not seldom injurious. Even since the commencement of this paper, we have seen several cases where patients were harrassed with local and general bleeding for pains in the head, the hypochondria, and the stomach, which turned out to be *neuroses*, and not *phlogoses*, after all! We need not say that, when a practitioner is obliged to veer round from venesection to bark and arsenic, the patient naturally enough asks himself how it is that the Doctor did not give that medicine at first which cured the disease at last. These are occasions in which we cannot always veil the mistakes which we make, and, therefore, we have the more need to look sharply into the character of the maladies we are treating.

We shall wind up this memoir with one or two of the general conclusions, or rather inferences, drawn by our author, and which appear to us to be well-founded.

"1mo. That which distinguishes the visceral from the external neuralgiæ, is the greater severity of the pain in the *latter* class, which generally comes on in the evenings; whereas the visceral neuralgiæ are worse in the morning, and become mitigated in the evening.

"2do. Both classes of these neuralgiæ have the following characters in common, namely, a *preceding* malaise, nausea, chilliness, some præcordial anxiety—an *accompanying* irritation, and afflux of blood, with or without febrile action— a termination in perspiration or sedimentous urine—and, finally, intermissions between the paroxysms. These neuralgiæ, in both classes of nerves, cede to the same therapeutical means—and generally leave no cognizable trace of their existence after death, either in the nerves themselves, or in the organs to which these nerves are distributed."—*Bib. Med. Juin*, 1828.

---

64. Dr. Long—Consumption.

Among the *hallucinations* of the day, is the wide-spreading fame of the above-mentioned Doctor (whose degree in medicine was, we suppose, conferred by the Man in the Moon) for the cure of phthisis! The Doctor's remedy is so invaluable, that we understand he causes the patient to swallow it in his own presence, lest any analysis should be made, and its nature ascertained. The Doctor's generosity is unbounded, for he gives the medicine "free gratis"—and merely charges the trifling sum of one guinea each day for the trouble of seeing it swallowed. The fame of the Doctor, like Fame of old, is reaching above the clouds—and all ranks, gentle and semple—or rather gentle and *simple*, are repairing to the learned Doctor for the cure of consumption! We have seen several of his cures, and also of his failures. The following case, which presented itself this very day (20th Aug.) will serve as a specimen of the *kind* of consumptions, and of the wonderful cures of that disease, which are so much bruited about—especially in that prostituted, venal, and talentless journal, the Literary Gazette.

A young man residing near Kensington, who had embarked in some business, and soon lost the whole of his little property, became despondent, dyspeptic, and *lost his flesh*. These were symptoms, especially the last one, which left no doubt in his own mind, or in that of his friends, that he was in a "*rapid decline*." The family practitioner failed to effect a cure—for alas, the cause was in the mind—and Dr. Long was applied to. The young man had neither cough, expectoration, fever, dyspnœa, or a single symptom of phthisis, save the *loss of flesh*—but this was quite enough, in the Doctor's eyes, to constitute "consumption," especially as the patient complained of wandering pains about the sides, shoulders, and pit of the stomach. What the internal medicine was on this occasion, we do not pretend to know; but, on stripping the chest, we found the whole surface covered with pustules produced by tartar-emetic. This powerful irritation on the surface had masked, or probably removed all the wandering pains; and it was confessed by the patient that Dr. Long had done him more good than the regular practitioner. But the emaciation went on, and, therefore, Dr. Long had failed to remove the main source of alarm. On examination by the stethoscope and percussion, the lungs were found to be perfectly sound. The tongue was foul, the epigastrium tender, the appetite bad, the digestion painful, the mind irritable and despondent, the urine sometimes pale, sometimes lateritious—in short, the patient was labouring under dyspepsia in an aggravated form, attended, of course, with *loss of flesh.* This was one of the cases of consumption which Dr. L. undertakes to cure—and we have no doubt that such cases constitute nine-tenths of those which—do *not die* under the Doctor's care. It is true he does not cure them; but, as they are considered to be in a galloping consumption, their not dying at the time, and their ultimate recovery by the powers of Nature, are signal triumphs of the Doctor's skill.

We should not have deigned to blot a page of our work with the notice of such an animal as Dr. Long, had we not been applied to by medical men in distant parts of the empire, who were importuned by the friends of patients to learn the medical character of this piece of effrontery. The present short notice is all that is necessary, as our readers will readily draw their own conclusions—and they can present enquiring patients with this portrait of the consumption-curer, if their own testimony is doubted.

We cannot, however, close this article without again expressing our indignant contempt of that sink of prostitution, the LITERARY GAZETTE. If, for a paltry premium, they have trumpeted forth the mendacious cures of consumption by such an impudent medicaster as Dr. Long, in order to deceive the unsuspecting sufferer and tamper with his pocket and his health, they deserve the reprobation of the public at large. If they have opened their columns, *through ignorance*, to designing knaves, who are paid for these fulsome puffs, they are scarcely less culpable—and not at all less despicable.

---

### 65. MELANOSIS.

Messrs. Trousseau and Leblanc have been making some extensive researches into this strange and dangerous disease, at the Veterinary School of Alfort. Melanosis is extremely common in horses—scarcely does a white or grey horse die without presenting more or less of this malady. The investigations of these gentlemen leave little or no doubt that melanosis is a morbid secretion, or rather a morbid condition of the blood. The following examination will support this opinion.

"On opening the chest of an old white horse, whose body presented innumerable melanic masses, we were surprised at the singular appearance which the pleura pulmonalis presented. It seemed completely marbled or speckled with blue, black, and violet spots. These spots sometimes united and formed groups, between which the pleura was of its ordinary colour. There was no saliency of the pulmonary tissue in these places. In these aggregations, the membrane appeared as if sprinkled with large drops of mulberry juice. The pleura was carefully detached, and spread upon glass. It was then examined by the aid of a good microscope. The membrane was every where transparent in the interstices; but where it covered the spots, it was slightly opake or faintly red. The spots appeared to be a kind of petechiæ—or rather an effusion of some colouring matter into the membrane and its subjacent cellular tissue. Not the least trace of vascularity could be discovered by the microscope—but simply an agglomeration of sanguineous globules, which, in the centres of the spots or stains, were too numerous to be distinct, but easily cognizable at the margins. The largest and deepest-coloured spots, the globules were perceived to be jet black. The pulmonary tissue was sound, except that, immediately opposite the pleural stains, it was rather inclined to hepatization."

M. Rigot has also remarked numerous ecchymoses on the serous membranes of horses presenting melanosis in other parts, and these ecchymoses he considers as the first degree of the malady. It appears to our authors, in fact, that blood is first effused, and that it subsequently becomes transformed into the melanic matter.

These depositions are not peculiar to any particular part or tissue of the body. They have been found pervading all the different structures. They do not exert that deleterious influence on the animal economy which scirrhous and fungoid tumours produce. It is only when they soften down, or when they enlarge to such a size as to incommode the functions of contiguous organs, that they become dangerous. The authors of this memoir have seen numerous horses which have presented very large masses of melanosis, and yet enjoyed good health to the last. —*Archives Générales.*

---

66. Remarkable Case of Spectral Illusion, apparently connected with some Local Morbid Activity of the Brain.

The following narrative (lately read at the London Phrenological Society) appears to be drawn up without any theory, and without any belief in the *reality* of the phenomena which presented themselves, not merely to the eye of the mind, but to the material organ of vision. The gentleman who records, and was the subject of, the spectral illusions, is a learned member of the English bar.

"In December, 1823, A. was confined to his bed by inflammation on the chest, and was supposed by his medical attendant to be in considerable danger. One night, while unable to sleep from pain and fever, he saw, sitting in a chair, on the left side of his bed, a female figure, which he immediately recognized to be that of a young lady who died about two years before. His first feeling was surprise, and perhaps a little alarm; his second, that he was suffering from delirium. With this impression he put his head under the bedclothes, and after trying in vain to sleep, as a test of the soundness of his mind he went through a long and complicated process of metaphysical reasoning. He then peeped out, and saw the figure in the same situation and position. He had a fire, but would not allow a candle or nurse in the room. A stick was kept by his side, to knock for the nurse when he required her attendance. Being too week to move his body, he endeavoured to touch the figure with the stick; but, on a real object being put upon the chair, the imaginary one disappeared, and was not visible again that night.

"The next day he thought of little but the vision, and expected its return without alarm, and with some pleasure. He was not disappointed. It took the same place as before, and he employed himself in observations. When he shut his eyes or turned his head, he ceased to see the figure; by interposing his hand he could hide part of it; and it was shewn like any mere material substance, by the rays of the fire which fell upon and were reflected from it. As the fire declined it became less perceptible, and as it went out, invisible. A similar appearance took place on several other nights, but it became less perceptible, and its visits less frequent, as the patient recovered from his fever.

"He says the impressions on his mind were always pleasing, as the spectre looked at him with calmness and regard. He never supposed it real; but was unable to account for it on any philosophical principles within his knowlege.

"In the Autumn of 1825, A.'s health was perfectly restored, and he had been free from any waking vision for nearly eighteen months. Some circumstances occured which produced in him great mental excitement. One morning he dreamed of the figure, which stood by his side in an angry posture, and asked for a locket which he usually wore. He awoke and saw it at the toilet, with the locket in its hand. He rushed out of bed, and it instantly disappeared. During the next six weeks its visits were incessant, and the sensations which they produced were invariably horrible. Some years before he had attended the dissection of a woman in a state of rapid decomposition. Though much disgusted at the time, *the subject* had been long forgotten; but was recalled by the union of its putrescent body with the spectre's features. The visits were not confined to the night, but frequently occurred while several persons were in the same room. They were repeated at intervals during the Winter; but he was able to get rid of them by moving or sitting *in an erect position*. Though well, his pulse was hard, and generally from 90 to 100."* 211.

---

* Phren. Journ No. XVIII. Aug. 1828.

The narrator is a gentleman of good education and literary habits who never considered the phenomena as any other than illusions. There are others, of a similar kind, on record; and they can only be accounted for by some local morbid action going on in the brain. It is needless to say that in a credulous person, the above phenomena would have produced a complete conviction of the *reality* of the figure which paid its nightly visits—in other words, of the reality of GHOSTS. Samuel Johnson would assuredly have believed in the reality of the apparition.

---

67. THE MIDLAND MEDICAL AND SURGICAL JOURNAL.

The appearance of a provincial Medical Journal is a novelty in this country, though not so on the Continent. It is not a little remarkable that Glasgow and Worcester should each send forth a quarterly medical journal, while all Ireland cannot, or rather *will not*, produce a single specimen of what is universally allowed to be a useful vehicle of professional information! We believe we could assign very probable causes for this circumstance, but we do not deem it necessary on the present occasion. Ireland presents an anomaly in *politics* as well as in *medical literature*, and we suspect that the *former* has more influence on the *latter* in the Emerald Isle than on this side of the Channel. Be this as it may, the birth of a provincial medical journal may fairly excite some reflexions. The general opinion, as far as we have been able to learn, is unfavourable to the success of such an undertaking; though, in no one instance, has the predictor been able to assign any valid reason—or, indeed, any reason at all, for this ominous prediction. We were pondering on the solution of this knotty question the other day, while walking past Charing Cross, that immense confluence of the various tides of human existence, when we cast our eyes on a splendid boot-shop, where it was emphatically announced that none but *town-made* boots were sold. On enquiry, we found that *boots* are infinitely better if they happen to be made in the smoke of London, than if manufactured in the clear air of the country. We asked the reason. There were no less than two reasons. The materials were better—the workmanship was superior. As a necessary consequence, the manufactured article was much dearer. Here then was some clue to the solution of the prejudice above-mentioned. If *town-made boots* are better than country-made articles, why should not *town-made books* be superior to those manufactured in the country? By such analogies and by such ratiocination, however absurd, the great majority of mankind are guided. But to reason a little closer. If it be true that medical science is founded on observation, then we cannot conceive why facts and observations recorded in the country, should not be as valuable as those recorded in the metropolis. It is true that the capital of a country attracts talents—but it also attracts every thing capable of alloying talent. If books are better composed in London than elsewhere, they are like the bread there—fair to the eye, but poisonous to the stomach. The MIDLAND REPORTER may not know—

> the city journal's art,
> Whose frothy pertness charms the vacant heart; nor open its venal columns as the channels of public calumny, to gratify personal spite. Its Editors have a "stake in the country"—they have characters to lose—and, therefore, they will not endeavour to degrade the profession to fill their pockets.

We have heard it said that the MIDLAND REPORTER cannot stand. We doubt the truth of this prediction. If it were set on foot as a pecuniary speculation, to extricate its conductors from the rules of the FLEET or KING'S BENCH, then we candidly confess our fears that the Editors would long remain in those dreary haunts. They are not the people calculated to "raise the wind" by tales of terror—midnight assassins—portentous conflagrations*—unnatural crimes—or revolutionary insurrections.† They write not for bread—they write not for gain—but for the good of their profession, and

---

* "Nocturnos lemures, portentaque Thessala, Terrores magicos."——HOR.

† By the way, where is the surgical *legion* that was to have overwhelmed the House of Commons with petitions, and stormed the citadel in Lincoln's-Inn-Fields? Not a voice responds to the plaintive notes of the tuneful instrument.

with the laudable wish to signalize themselves by their contributions to medical science. These objects will be obtained by even a very limited circulation of the Midland Reporter, since the contents of the work will be dispersed through various channels, over every country which—

"The rising or the setting sun surveys."

It will be a shame for the midland counties—for the profession at large, if three or four hundred subscribers cannot be found to pay the primary expences, beyond which nothing else is sought or expected. Every book-society in the kingdom should encourage a work, whose sole object is to report such useful facts as occur in hospital or private practice. These unassuming labours *ought to be* acceptable to all parties—to all ranks of the profession—and we sincerely hope they *will* prove so. In the mean time, we hesitate not to say, that we have felt some disappointment in the first No. of the work under consideration. The Essays on "Medical Topography" promise to be too prolix, and excursive into regions of investigation that have little connexion with the subject. Neither do we think that the article on fever is exactly suited for publication, however well it might have been adapted for the occasion which first gave it birth. But it is not fair to judge by "the first appearance on any stage," and we have no doubt that the Midland Reporter will soon take a respectable station among the medical journals of the day.

---

68. Enormous Hypertrophia Cordis—Disease of Semilunar Valve—Diseased Structure of Liver—Œdema of the Mucous Membrane of Stomach and Bowels, &c.

We are sorry to record the death of a medical gentleman, in the prime of life, or rather of youth, and whose talents and amiable character promised to render him an ornament to our profession. Dr. Cox, author of a Treatise on Rheumatism, a Dispensatory, &c. went out, about three years ago, to the Caraccas, as physician to our Consul there, Sir Robert Kerr Porter, and returned from thence about the beginning of November, 1827. Shortly after his arrival he was seen by the Editor of this Journal, who examined his chest with attention. The action of the heart was excessive—the organ beat over a large space—the arteries throbbed violently—the jugular veins pulsated—the "*bruit de scie*" was very distinctly heard—the respiratory sound was audible in all parts of the chest, which also sounded well on percussion. Dr. Cox appeared very much agitated and nervous; it was, therefore, hoped that part, at least, of the great action of the heart was attributable to nervous palpitation. The countenance was pallid, and inclined to be sallow—the breathing was disturbed by exertion, especially by ascending stairs or high ground. In other respects, there was no apparent disorder of any organ in the body. Each subsequent examination tended to increase the fear, that active enlargement of the heart was going on, and, in this opinion, Dr. Clark, and several other medical friends, concurred. It was rather remarkable, however, that two or three glasses of wine invariably lessened the action of the heart, and ameliorated the patient's distressing feelings—especially the pain in the region of the heart, of which Dr. C. often complained. Local depletion, counter-irritation, digitalis and diuretics, were long and vigorously employed, but not, we believe, with any decided effect. Dr. J. lost sight of the patient for seven or eight months, and did not see him till the 10th of August, ten days before the fatal termination of the disease. Dr. Cox called on Dr. Johnson, and stated that his complaint had recently taken an entire new turn. The distress in the region of the heart was greatly mitigated, and his principal sufferings were referred to the epigastrium, which was so exquisitely tender, and withal so prominent, that a physician that morning thought there was acute inflammation and enlargement of the liver. On examination of the chest,

---

The lyre is hung on the weeping willow—and sighs to the passing winds, while deploring the ebbing tide of human passions, no more to be hurried along under the influence of another Lucifer. In thus turning a deaf ear to the declamations of self-interest, the profession has shewn symptoms of penetration, that are not lost or unperceived in the proper quarter!!

there did not appear to be any change in the physical signs of hypertrophy of the heart—on the contrary, they seemed all to have increased. But the tenderness and pain in the epigastrium (where, by the way, there was a tremendous pulsation,) masked the other feelings, or engrossed Dr. Cox's entire attention. Leeches, blisters, two general bleedings, gave little or no relief. The breathing became so embarrassed that he was obliged to be propped up in bed, and he expressed himself as always on the brink of suffocation. In this dreadful state he lingered till Wednesday, the 20th August, when death put a period to his sufferings.

The body was examined by Dr. Hodgkin, in presence of Mr. Morrah, Dr. Johnson, and two medical students. The surface was slightly tinged yellow, and the ankles were œdematous. In the chest there were better than three pints of reddish serum, but no marks of inflammation. The heart was so enormously enlarged, that neither Dr. Hodgkin, Mr. Morrah, nor Dr. Johnson, had ever seen one of such a size before. The cavities were dilated as well as the parietes thickened. There was nothing the matter with the mitral or tricuspid valves; but one of the semilunar valves of the aorta was dilated into a pouch that would receive the end of the thumb up to nearly the first joint. The inner coat of the aorta, opposite to this valve, was thickened and irregularly raised. There was no other morbid appearance in the arterial system. The lungs, especially on the left side, were condensed, but not like hepatization from inflammation. In the upper lobe of the right lung was found some small masses of calcareous deposit, and the remains of a tuberculous excavation, completely lined with a shining membrane, and communicating with one of the bronchia. The liver was rather smaller than usual, hard, and curiously mottled with yellow and red specks, so as to present an extremely diseased appearance. The spleen was very much indurated, and carnified. But the most remarkable phenomenon in the abdomen was the state of the mucous membrane of the stomach and intestines. Besides a very minute injection of the vessels, the membrane was, in a very great extent of surface, completely *œdematous*. The rugæ in the intestines presented the appearance of bladders filled with a thick but pretty clear fluid, as in some blisters.

Dr. Hodgkin was of opinion that the state of the semilunar valve in the aorta, as above detailed, might be the cause of the hypertrophy, as not only keeping up irritation in the neighbourhood of the ventricle, but as checking the issue of blood from the left side of the heart. The writer of this notice cannot entirely coincide with Dr. H. on this point. The action of all tangible arteries was in proportion to that of the heart, and there did not appear to be the least check to the issue from the ventricle into the aorta.* The patient had had acute rheumatism, and this, combined perhaps with a faulty constitution from birth, may have determined the disease of the heart. The dissection was interesting in another point of view. It shewed the natural cure of a tuberculous excavation—in short, Dr. Cox must, at one time, have had tuberculous expectoration, or, in other words, phthisis. Considering that two if not three of his brothers died of phthisis, it is remarkable that a broken down tubercle should have terminated favourably in such a constitution. There were no other tubercles, however, in the lungs. The patient bore his dreadful sufferings during the last ten days of his life, with heroic firmness of mind, although he was conscious of the fatal nature of the malady!

---

### 69. SENSES OF SMELL AND SIGHT IN CERTAIN BIRDS OF PREY.

It always appeared to us most extraordinary—indeed unaccountable, that birds of prey could *scent* carcasses at such im-

* The rush of blood, however, past this enlarged valve, at each stroke of the ventricle, was evidently the cause of the "BRUIT DE SCIE," or "stroke of a saw," which was heard corresponding with the pulse and the ventricular contractions. It is difficult to conceive the cause of this enlargement of the valve in a direction *contrary to the current* of the blood. The only way in which it can be accounted for, is a frequent attempt at *retrogradation* in the circulation through the aorta, as it is only then that the semilunar valves can be put on the stretch.

mense distances as they are said to do. We were led to scepticism on this subject, some 20 years ago, while observing the concourse of birds of prey *from every point of the horizon*, to a corpse floating down the River Ganges—and that, during the north-east monsoon, when the wind blew steadily from one point of the compass for months in succession. It was extremely difficult to imagine that the effluvia from a putrefying body in the water could emanate in direct opposition to the current of air, and impinge on the olfactories of birds many miles distant. Such, however, were the DICTA of natural history, and we could only submit to the general opinion. We have no doubt, (now that we know general opinion to be sometimes wrong,) that it was by means of the optic rather than the olfactory nerves, that the said birds "smelled out their suit." The TOUCAN is a bird which ranks next to the vulture in discerning (whether by sight or smell) the carrion on which it feeds. The immense size of its bill, which is many times larger than its head, was supposed to present, in its honey-comb texture, an extensive prolongation of the olfactory nerve, and thus to account for its power of smelling at great distances. But, on accurate examination, the texture above-mentioned in the bill is found to be mere diploe to give the bill strength. Now the *eye* of this bird is somewhat larger than the *whole brain*, and it has been ascertained by direct experiments, that, when very putrid carrion was inclosed in a basket, from which the effluvia could freely emanate, but which concealed the offal from *sight*, it attracted no attention from vultures and other birds of prey, till it was exposed to their *view*, when they immediately recognized their object, and others came rapidly from different quarters of the horizon, where they were invisible a few minutes before. This sudden appearance of birds of prey from immense distances, and in every direction, however the wind may blow, is accounted for by their soaring to an altitude far beyond our sight. In this situation, their prey on the ground is seen by them, however minute it may be, and therefore their appearance in our sight is merely their descent from high regions of the atmosphere to within the scope of our optics. The toucan in India generally arrives a little in the rear of the vulture, and remains till the larger bird is glutted—while smaller birds of prey, at a still more retired distance, pay similar homage to the toucan!

---

### 70. Cases of Retroversio Uteri. By William Weir, M. D.

In the last number of our Glasgow Cotemporary, Dr. Weir, one of the surgeons to the City poor, has published two cases of this complaint, which we shall here notice. They both terminated favourably, and both the females are now living.

Dr. W. conceives that, although pregnancy is not essentially necessary for the occurrence of this accident, yet some enlargement of the uterus must have preceded the retroversion. It generally takes place about the third or fourth month of utero-gestation. At this period, more particularly in women who have the brim of the pelvis rather contracted, and the general cavity wider than natural, the fundus is liable to fall backwards upon the rectum, the os uteri being turned upwards upon the symphysis pubis.

*Case* 1. "The first was a married woman, aged 48, the mother of ten children, the youngest five years old. I was called to her on the 6th November, 1818. She had been complaining for about a fortnight, of occasional severe pains in the region of the bladder and uterus, stretching down the thighs, and coming on "in showers" similar to labour-pains, but often more severe. She could not pass urine but in drops, had a constant desire to go to stool, with much straining and distressing tenesmus, but very scanty evacuations. The distended urinary bladder was distinctly felt above the pubes. She had not menstruated for three months, though previously regular, and she attributed her complaints to extreme costiveness, occasioned, as she thought, by improper diet, and producing excessive straining every time she went to stool. Three pounds of urine were drawn off by the catheter, with considerable relief. The instrument being in some degree obstructed, while passing into the bladder, led me to examine the vagina, when I discovered a hard tumour lying in the hollow of the sacrum, between the rectum and bladder. Firm pressure produced considerable pain, and great inclination to go to stool. The os uteri was discovered lying close upon the symphysis pubis, pressing upon the urethra. This tumour could also be felt by the finger introduced into the rectum. Fourteen ounces of blood were taken from the arm, a purgative enema exhibited, and fomentations ordered to the abdomen. In the evening the catheter could not be introduced, the injection had produced no effect, she had passed no urine, and next day, (7th,) she was greatly worse, with much thirst, foul tongue, and pulse 100. The instrument was now introduced with some difficulty, and the bladder emptied. Purgative enemata, with occasional doses of calomel and jalap, procured some evacuation from the bowels, and the urine was drawn off morning and evening. The warm bath, with leeches to the vulva, had the effect of relieving the severe tenesmus and distressing bearing-down efforts. On the 12th, while in the bath, she succeeded in passing a great quantity of urine, and some hours afterwards, she again made as much, after which, the symptoms became more manageable and gradually gave way. On the 16th, she had a copious and formed stool for the first time since her illness; the previous evacuations from the bowels having been all along either liquid, or of a flattened form, as if the sides of the intestine had been kept nearly in contact by the tumour. For some time after this, she was occasionally troubled with tenesmus, and had a more frequent desire than natural to pass urine, which symptoms were relieved by opiate injections. In a few weeks, she got quite well, and is at present in good health."

In this case there was no pregnancy; but Dr. W. believes there was enlargement of the womb, the patient being about the change of life.

*Case* 2. This was a most formidable one, and nearly proved fatal. The patient was a widow, aged 39, and had borne several children. She firmly denied pregnancy. She complained of the same symptoms described in the preceding case —but especially of the severe bearing down pains. She had been ill for three weeks—and had taken strong purgatives, (for the purpose of procuring abortion, as was afterwards ascertained,) that operated violently. Her urine has been dropping away involuntarily for two or three days—the belly was swollen, and immediately above the pubes there was a firm tumour, painful on pressure.

"The finger, introduced into the vagina and directed towards the os coccygis, was prevented going far, by a hard tumour lying in the hollow of the sacrum. On turning the finger upwards before the symphysis pubis, the same tumour was felt pressing upon the urethra, and preventing the free evacuation of the urine. It was also discoverable by the rectum, keeping the sides of that intestine together, so as nearly to obliterate its cavity. After the most careful examination, the os uteri could not be discovered in any direction. She had not menstruated for three months, but denied being pregnant. The catheter was introduced without much difficulty, and 2lbs. of urine drawn off. An ounce of castor oil was given, and an anodyne draught ordered at bedtime. Next day, 1st November, there was no change for the better; the physic had operated, the stillicidium urinæ continued, and 4lbs. of urine were drawn off by the catheter. The swelling of the abdomen appeared considerably less, but there was still a hard firm tumour immediately above the pubes; and although no more urine would flow, I did not consider the bladder completely emptied. There was now considerable œdema of the legs, thighs, and lower part of the belly. On the 2d November, the tumour was felt nearer the orifice of the vagina, and the distressing tenesmus and bearing-down efforts were more severe. The stillicidium continued, the belly was much swoln, and the bladder seemed full of urine. I experienced considerable difficulty in introducing the catheter. It went in a certain way easily, and about 2lbs. of urine flowed through; but although it was quite evident that much remained in the bladder, no more could be obtained, and the tumour prevented the instrument passing any farther up. Elastic gum and silver catheters, male and female, of different sizes, were tried, the patient being placed in various positions, but they were stopped after being introduced about three inches.* A small

* "Mr. Syme, who relates a case of quantity of laudanum was occasionally thrown into the rectum, which relieved the irritation; but a purgative injection which was this day prescribed, could not be got in. I had now the assistance of my friend Mr. Stirling in the management of the case, and several other medical gentlemen occasionally visited her. On the 3d and 4th, she continued much in the same way; but the introduction of the catheter, which was attempted morning and evening, gradually became more difficult, less urine could be got away, and the abdomen increased in size almost every hour. On the 6th, the bladder was felt considerably above the umbilicus, and the fundus of the womb was getting lower in the pelvis. The catheter could still be introduced three inches, and today 2 lbs. of urine came away, but with little diminution in the size of the abdomen."

It was proposed to puncture the bladder above the pubes, but the patient would not consent. Things got worse and worse, till the following deplorable condition obtained.

"On the morning of the 9th November, the swelling of the abdomen was much increased, and she had paroxysms of severe pain almost constantly. The catheter brought away only four ounces of urine. The anasarca of the limbs and integuments of the abdomen, had increased greatly. There was considerable fever, with great thirst, furred tongue, and quick pulse. About mid-day, she was seized with the most severe pains, which continued almost without intermission, and were evidently those of labour. A consultation was called at five o'clock, P. M. On examining the state of the parts, we found the tumour now lying quite at the entrance into the vagina. The uterus appeared to be almost turned upside down; it lay impacted between the rectum and bladder, and it was with much difficulty that any examination could be made, owing to the strong action of the abdominal muscles forcing the contents of the pelvis downwards, and preventing the introduction of the fingers into the vagina. The pains were constantly present, and it appeared that the uterus was endeavouring to expel its contents, but which could not be accomplished while the os uteri remained in its unnatural situation. There was acute fixed pain in the back and at the bottom of the abdomen, immediately above the pubes. All the gentlemen were of opinion, that if some relief were not obtained, the patient could not survive long. There was now no hope that the uterus could replace itself. The action tended all the other way. The strong muscular exertions of the patient forced the fundus still lower and lower. The question was, What could be done for her relief? Puncturing the bladder above the pubes was now considered quite inadmissible; inasmuch as merely emptying the bladder would have little effect in bringing down the mouth of the womb, and could not be expected to render any manual operations more easy, or more likely to be successful. One gentleman proposed that the fundus of the uterus should be punctured, which would allow the liquor amnii to escape, and reduce very materially the size of the tumour. Previous to deciding upon this proposal, it was agreed to make a last attempt to reach the os uteri, more particularly as Mr. Stirling believed, that at a former examination, he had touched with his finger the posterior lip lying high up above the pubes. Accordingly, after much difficulty, and a great degree of force, and in opposition to the strong and powerful exertions of the patient, which all tended to prevent its admission, I succeeded in getting my hand into the vagina, forced up my finger above the pubes, and reached the mouth of the womb. An assistant at the same time got his hand into the rectum, and we had thus the perfect command of the patient. By steadily pushing upwards the fundus, and cautiously pulling the neck and mouth of the womb downwards, the tumour was gradually raised above the promontory

---

Retroversion in the Medical Observations, (vol. iv. p. 392), mentions, that the catheter was with difficulty introduced "an inch or two," and afterwards it seemed to pass rather *farther up the urethra*, but only drew off a spoonful or two of urine.

"In Dr. Cheston's patient, he had to introduce a long male flexible catheter half its length, before any urine came away; and even after it was in three-fourths of its length, he did not draw off 'such a quantity of urine as sensibly to diminish the swelling of the abdomen.'"—Medical Communications, vol. ii.

of the sacrum, and the uterus reduced to its proper position. Considerable quantities of urine had been evacuated during the operation, and previous examinations. The labour continued; and now, after the uterus was freed from its firmly impacted state, the pains assisted to bring it still more perfectly into its natural situation.

"It was ascertained that the membranes continued unbroken, the os uteri being little dilated; but after a few pains, the waters came away, and a leg and thigh were protruded. The abdomen had fallen greatly, was much softer, still tender when pressed upon, and the firm tumour, formerly observed immediately above the pubes, had now disappeared. The os uteri was not much dilated; and although the labour pains continued pretty strong for some time, they afterwards fell off, and the fœtus made no progress for twenty-four hours. At this time she was seized with a severe rigor, and strong bearing-down efforts. A medical gentleman in the neighbourhood saw her immediately, and thought it proper to extract the fœtus, the os uteri being then well dilated. The child was brought away mutilated; it was apparently about the fourth month, and somewhat putrid. The patient had passed urine naturally several times since the replacement of the uterus."

Severe abdominal inflammation supervened, and was checked by vigorous depletion. She perfectly recovered. The above case is very creditable to the courage, dexterity, and skill of the medical attendant. It may suggest some useful hints to our obstetric brethren.

---

71. Cases of Organic Disease (several Tubercles) of the Cerebellum. By M. Rennes, Physician to the Military Hospital of Strasburgh.

The functions of the cerebellum have, of late years, caused much discussion among physiologists and phrenologists, and many a poor animal's brain has been sliced to determine the relative functions of parts, without deciding the questions at issue. It is probably by morbid anatomy, after all, that we shall be able to localise the organs, and assign to them their proper uses and powers. The following case is interesting in a simple pathological point of view, and we shall briefly record it.

*Case.* A young soldier, aged 21 years, of slender constitution, was admitted into the Military Hospital of Strasburg, on the 1st of January, having been two months in the Hospital of the Royal Guard. M. Rennes now watched the case narrowly, day by day. The patient complained of acute and deep-seated pain in the back part of the head—sleeplessness—debility. He was greatly emaciated, and vomited his food—lay on his right side in bed—was morose and taciturn—and complained reproachfully of the inefficacy of medicine in his case. The pulse was small and frequent—skin dry and harsh—eye hollow and expressive of great suffering—tongue natural—no appetite—slight uneasiness at the epigastrium—most indomitable constipation. These symptoms, especially the pain in the occiput, and the vomiting of every thing taken, continued in despite of leeches, blisters, &c. and death put a period to the patient's sufferings on the 16th February.

*Dissection.* There was some water in the ventricles of the brain, which was much softened in its structure. There was one large tubercle in the left lobe of the cerebellum, and several in the right lobe. The substance of the cerebellum was healthy, even in the immediate neighbourhood of the tubercles. There were some traces of inflammatory action in the mucous membrane of the stomach.

The above case has been brought forword by M. Rennes, in opposition to phrenology—though in what way it can afford matter of argument for the antiphrenologists, we are at a loss to imagine. Tubercles were developed in the cerebellum we allow—but the intervening substance of that organ was healthy. Surely the cerebellum, in such circumstances, might carry on its functions as well as the lungs when tuberculated. But we have noticed this case on account of the obstinate vomiting which evidently was dependent on the cerebellic affection. We have seen three instances where vomiting was the most prominent and distressing feature of the complaint during life, and where dissection shewed no disease of the stomach, but tubercles in the cerebellum. The case lately published by Dr. Chambers, from St. George's Hospital, is another example of the same kind.

### 72. REPORT OF THE ANATOMICAL COMMITTEE.

The following highly interesting document deserves permanent record on our pages, as marking an important æra in the progress of medical science—an æra, in which the light of reason and truth has penetrated into the darkest recesses of the human mind, and begun to dissipate its most rooted prejudices.

### REPORT.

The peculiar nature of the subject which the Committee were appointed to investigate, has induced them to inquire principally into the practice of the anatomical schools of London, where, by personal communication with the most eminent surgeons and with the students and principal teachers of anatomy, it could be fully ascertained that no detriment to their interests was to be apprehended from the publicity to arise out of the present inquiry. With regard to the practice of the provincial schools, to avoid the expense of summoning witnesses from a distance, they have been satisfied with written communications from resident professors or practitioners of eminence, which will be found in the Appendix.

The Committee have inquired into the nature of the difficulties which the anatomists have here to contend with, whether arising out of the state of the law or an adverse feeling on the part of the people; and into the evil consequences thence ensuing, as well to the sciences of medicine and surgery as to all who study, teach, and practise them, and eventually to the members of the whole community. They have called witnesses to shew in what manner the wants of the anatomist are provided for in several foreign schools, and to state their opinion whether similar methods could be applied with advantage in this country, and if applied would be adequate to remove the present difficulties.

The first origin of these difficulties is obviously to be traced to that natural feeling which leads men to treat with reverence the remains of the dead; and the same feeling has prompted them, in almost all times and countries, to regard with repugnance and to persecute anatomy.

As the importance of the science to the well-being of mankind was discovered, the governments of different states became its protectors, and in this country particularly, by the statute of Henry VIII. protection to a certain extent was given and intended to be given to it; but that protection, which at first perhaps was fully adequate, owing to the rapid progress of the science, has long since become wholly insufficient.

How limited were the wants of the science in the former part of the last century may be learned from the lectures of Dr. William Hunter, who describes the professors of the most celebrated schools, both at home and abroad, as employing in each course of lectures not more than one, or at most two subjects, and as exhibiting the performance of the operations of surgery, not on human bodies, but on those of animals. He represents the students in medicine and surgery as never exercising themselves in the practice of dissection, because for such practice they had no opportunities.

For such a system of instruction the provisions of the statute of Henry VIII. might well be adequate, and these provisions, indeed, may now be considered of importance only as a distinct admission of the principle, that the government of this country ought to protect anatomy. The reformation of this antiquated and imperfect system took place in this country in the year 1746, when Dr. William Hunter, having a singular enthusiasm for the science, established complete courses of anatomical lectures, and opened a regular school for dissection. The reform thus introduced was complete, and its author exulted before his death in having raised and diffused such a spirit for dissection that he should leave behind him many better anatomists than himself.

Under his immediate pupils and their successors this school has gone on increasing. The earliest account that the Committee have met with of the number of anatomical students resorting to London, is that given by Mr. Abernethy, who states that shortly after the breaking out of the war with France they amounted to 200. One of the witnesses, Dr. Macartney, computes their number in the year 1798 at 300; and Mr. Brookes, a teacher of anatomy, in a calculation submitted to Sir Astley Cooper in the year 1823, then reckoned their number to be 1000. It appears from the returns now furnished by the teachers of the different schools in London, that their number at present is somewhat above 800; the diminution in the number since the year 1823 being the consequence, probably, of the pupils resorting to foreign schools, the advantages of which were less known at the former period than they are at present.

When it is considered what a demand there is for practitioners, as well to meet the wants of an increased population at home as of an extended empire of colonies and dependencies abroad, this rapid increase of students will not appear surprising; and if it is considered also that not only is that demand an increasing one, but that every practitioner, however humble, from that laudable desire for intellectual improvement which characterizes the present age, endeavours, if he can afford it, to obtain a good education, and must regard himself as ill educated if he has not gone through a course of dissection, the eventual increase of dissecting students can hardly be calculated, should their wants be supplied abundantly and at a cheap rate.

Although the students now attending the schools of anatomy in London exceed 800, not more than 500 of this number actually dissect. The duration of their studies in London is usually sixteen months, and during that time the number of subjects with which every student in surgery ought to be supplied appears from the evidence (although there is some difference on this point) to be no less than three; two being required for learning the structure of the parts of the body, and one the mode of operating. The total number of subjects actually dissected in the schools of London in one year, is stated to be not greater than from 450 to 500, which is after the rate of less than one subject for each dissecting student; a proportion wholly insufficient for the purposes of complete education.

Dissection on an extended scale began in this country before there existed any such general feeling in its favour, founded on an opinion of its utility, that the British government, after the example of some foreign governments, would venture openly to patronize it. Accordingly, when in 1763, Dr. Hunter proposed to build an anatomical theatre, and to endow it with his museum and a salary for a professor, provided the government would grant him a site of ground for the institution, and his late Majesty would extend to it his countenance and protection, he met with a silent refusal. It was therefore only by stealth, and by means not recognized by the law, that the teacher was enabled to procure subjects. These means, it is notorious, from the time of Dr. Hunter down to the present time, have been principally disinterment; though of late other illegal modes and contrivances, such as stealing before burial, personation of relatives for the purpose of claiming bodies, &c. have occasionally been had recourse to. For some time after the first establishment of dissecting schools, while the number of teachers and students was small and the demand for subjects very limited, the means which were resorted to for obtaining a supply were adequate to the wants of the students, and bodies were obtained in abundance and cheaply. The exhumators at that time were few, and circumspect in their proceedings; detection was rare, the offence was little noticed by the public, and was scarcely regarded as penal; so that (according to one of the witnesses) long after the decision of the judges, in 1788, that disinterment was a misdemeanor, prosecutions for this offence were not common, and offenders taken in the fact were usually liberated. If this state of things had continued, though the illegality of the practices had recourse to must be conceded, yet they could scarcely be said to occasion evils of such magnitude as to require a legislative remedy. But the number of students and teachers having greatly increased, and with them the demand for subjects and the number of exhumators, detections became frequent, the practice of exhumation notorious, and public odium and vigilance were directed strongly against the offenders. It may be collected from the debates in Parliament which took place in the year 1796, during the progress of a bill for subjecting to dissection the bodies of felons executed for burglary and robbery, that even at that time the public regarded disinterment with strong feelings of jealousy.

In proportion as the public became vigilant, the laws relating to sepulture were interpreted and executed with increasing rigour; and as the price of subjects rose with the difficulty of obtaining them, the premium for breaking the laws increased with the penalty. The exhumators increased in number, and being now treated as criminals, became of a more desperate and degraded character.

The parties of daring men who now took to raising bodies, did it happen (as was frequently the case) that, while in pursuit of the same spoil, they fell in with another, actuated by vindictive feeling, and regardless of the caution and secrecy on which the successful continuance of their hazardous occupation must depend, had contests in the places of sepulture, left the graves open to public gaze, or gave information to magistrates, or the relatives of the disinterred, against their rivals. Frequently, with a view to raise the price of subjects, to extort money, or to destroy rivalry, they have proceeded to acts of outrageous violence, tending to excite the populace against the teachers of anatomy. These, and similar acts of violence or imprudence, have been constantly bringing exhumation to light, and have exasperated the public against both the exhumator and the anatomist; and this to such a degree, that of late, in many cases, individuals, out of solicitude to guard the dead, have taken upon themselves to dispense with the laws of their country, and have fired upon parties attempting disinterment. Other circumstances, but of minor importance, have been assigned by some of the witnesses as augmenting the difficulty of obtaining subjects in London, or increasing the demand for them; but as regards them, the Committee beg leave to refer to the evidence itself. The general result has been, with some difference, according to differences of place and season (sometimes owing to the caprice and mercenary motives of the agents employed, at other times owing to the real difficulty of obtaining a supply), that of late subjects have been to be procured, either not at all, or in very insufficient quantity, and at prices most oppressive to the teacher and student.

The price of a subject, about thirty years ago, was from one to two guineas; the teacher now pays from eight to ten guineas; and the price has risen even to sixteen guineas. The teachers deliver subjects to their dissecting pupils at a lower price than that at which they purchase them, having been compelled to resort to this expedient, lest dissection in London should be abandoned altogether. The loss which they sustain is made good out of the fees which they receive for attendance on their lectures in the anatomical theatre. The cost of providing subjects is also enhanced to the teacher, by his being required occasionally to defend the exhumator against legal prosecution, and to maintain him against want, if sentenced to imprisonment, and his family, in case he has one, until the period of his punishment expires.

Nor is it only of a precarious, insufficient, and expensive mode of obtaining subjects that the cultivators of anatomy complain,—it is by the law, not as regards the exhumators, but as it affects themselves, that they are aggrieved.

The first reported case of a trial for disinterment is that of Rex *v.* Lynn, in the year 1788, when the Court of King's Bench, on a motion for an arrest of judgment, decided it to be a misdemeanor to carry away a dead body from a church-yard, although for the purpose of dissection, as being an offence *contra bonos mores* and common decency. In this state the law on the subject of disinterment, as interpreted by the Court of King's Bench, appears to have remained until the present year; when Davis and another were tried and convicted at the assizes at Lancaster, and subsequently received the sentence of the Court sitting at Westminster, for having taken into their possession, with intent to dissect, a dead body, at the time knowing the same to have been unlawfully disinterred. A respectable teacher of anatomy, residing at Liverpool, had been tried and found guilty on a similar indictment at the quarter sessions at Kirkdale, in the month of February in the same year. With these exceptions, magistrates appear hitherto to have taken no cognizance of receiving into possession a dead body, unless there were strict evidence that the receiver was a party to the disinterment; and on this practical view of the state of the law professional men also appear hitherto to have acted. At present, however, a most intelligent magistrate, one of the witnesses, considers that very slight evidence would connect the receiver with the disinterment; and that the purchase from the exhumator would suffice to send the case to a jury, the knowledge of the fact of disinterment being to be collected from the circumstances, if strong enough to justify the inference. It is stated that there is scarcely a student or teacher of anatomy in England who under the law, if truly thus interpreted, is not indictable for a misdemeanor.

According to the opinion of the last-cited witness, to be a party to the non-interment as well as to the disinterment of a dead body, would render a person indictable for a misdemeanor. Two cases are cited in support of this opinion. In the one, Rex *v.* Young, a non-reported case, but referred to by the court in the case of Rex *v.* Lynn, the master of a workhouse, a surgeon, and another person, were indicted for and convicted of a conspiracy to prevent the burial of a person who had died in the workhouse. In the other, Rex *v.* Cundick, which occurred at the Surrey Spring assizes in the year 1822, the defendant was found guilty on an indictment for a misdemeanor, charging him with not having buried the body of an executed felon entrusted to him by the gaoler of the county for that purpose; but with having sold the body for lucre and gain, and for the purpose of being dissected: and on this trial it was not considered necessary to prove that the body had been sold for lucre or for the purpose of dissection. The witness infers, from the analogy of all these cases, that to treat a dead body as liable to any thing but funeral rites, is an offence *contra bonos mores,* and therefore a misdemeanor.

This state of the law is injurious to students, teachers, and practitioners, in every department of medical and surgical science, and appears to the Committee to be highly prejudicial to the public interests also.

It is the duty of the student to obtain, before entering into practice, the most perfect knowledge, he is able, of his profession; and for that purpose to study thoroughly the structure and functions of the human body; in which study he can only succeed by frequent and repeated dissection. But his wants cannot adequately be supplied in this country, except at an expense, amounting nearly to a prohibition, which can be afforded only by the most wealthy, and precludes many students from dissecting altogether. From the precariousness or insufficiency of the supply, the dissections and lectures are often suspended for many weeks, during which the pupils are exposed to the danger of acquiring habits of dissipation and indolence; and, from the same causes, that important part of surgical education is usually omitted, which consists in teaching how to perform on the dead body those operations which the student may afterwards be required to practise on the living. But not only does the student find dissection expensive and difficult of attainment; but he cannot practise it, without either committing an infringement of the law himself, or taking advantage of one committed by others. In the former case, he must expose himself to imminent hazard, and in either he may incur severe penalties, and be exposed to public obloquy. The law, through the medium of the authorities entrusted with conferring diplomas, and of the boards deputed by them to examine condidates for public service, requires satisfactory proof of proficiency in Anatomical Science, although there are no means of acquiring that proficiency without committing daily offences against the law. The illegality and the difficulties attending the acquisition of the science, dispose the examiners in some cases to relax the strictness of their examination, and induce them, in the case of the Apothecaries' Company, to dispense with dissection altogether; the persons to whom certificates are granted by the examiners of this Company being those who, from their numbers* and extensive practice, ought especially, for the safety of the public, to be well instructed. The annual number of certificates so granted exceeds 400.

The teacher of anatomy, besides the evils which befal him in common with the student, has to suffer others, arising also out of the state of the law, which affect him with peculiar hardship. The obstacles which impede the study of anatomy in this country are such, and the facilities presented to the study in foreign countries are so great, that those English students who are desirous of obtaining a thorough knowledge of the science, desert the schools at home, and repair to those abroad. Their principal resort is to Paris, where 200 English students of anatomy are now pursuing their course of instruction. Dissection probably, under these circumstances, would scarcely be followed at home, were it not for the regulations of the College of Surgeons, which require the candidates for the diploma of the college to have learned the practice of surgery in a recognized school within the United Kingdom; so that the student, during the period required for learning this practice, in order that he may the sooner become qualified for his profession, employs a part of his time in learning also to dissect. These disadvantages, affecting the teacher, are such, that except in the most frequented schools, attached to the greater hospitals, few have been able to continue teaching with profit, and some private teachers have been compelled to give up their schools. To the evils enumerated it may be added, that it is distressing to men of good education and character to be compelled to resort, for their means of teaching, to a constant infraction of the laws of their country, and to be made dependent, for their professional existence, on the mercenary caprices of the most abandoned class in the community.

But it is not only to the student, while learning the rudiments of the science, and to the teacher, while endeavouring to improve it, that dissection is necessary, and the operation of the law injurious. It is essential also to the practitioner, that during the whole course of his professional career he should dissect, in order to keep up his stock of knowledge, and to practise frequently on the dead subject, lest, by venturing to do so unskilfully on the living, he expose his patients to imminent peril. He is required also, in many important cases, civil and criminal, to guide the judgment of judges and of jurors, and would be rebuked were he to confess, upon any such occasion, that, from having neglected the practice of dissection, he was unable to throw light upon a point at issue in that science which he professed. He is liable, in a civil action, to damages for errors in practice, due to professional ignorance; though at the same time he may be visited with penalties as a criminal, for endeavouring to take the only means of obtaining professional knowledge.

Under these circumstances, affecting equally the student, teacher, and practitioner, the committee were not surprised to find that this inquiry excited considerable interest in all parts of the country, and that numerous petitions from all classes of the profession, connected with the science of anatomy, were laid upon the table of the house, uniformly praying for an amendment of the existing law on the subject.

But independently of the bearings of the question on the interests of medical practitioners, and on the health of the community, the system pursued is productive of great

* Computed at 10,000 in England and Wales.

evil, by training up a race of men in habits eminently calculated to debase them, and to prepare them for the commission of violent and daring offences. The number of persons who, in London, regularly live by raising bodies, is stated by the two police officers, examined before the committee, not to exceed ten; but the number of persons, occasionally employed in the same occupation, is stated by the same witnesses to be nearly 200. Nearly the whole of these individuals, as is admitted by the exhumators themselves who were examined before the committee, are occupied also in thieving, and form the most desperate and abandoned class of the community. If, with a view to favour anatomy, exhumation should be allowed to continue, it appears almost a necessary consequence that thieves also should be tolerated. It should seem useless, however, with a view to suppress exhumation, to endeavour to execute the existing laws with increased severity, or to enact new and more rigorous ones. The effect of interpreting and executing the laws with increasing rigour has been, not to suppress exhumation, but to raise the price of bodies, and to increase the number of exhumators. So long as there is no legalized mode of supplying the dissecting schools, so long the practice of disinterment will continue; but if other measures were devised, which would legalize and ensure a regular, plentiful, and cheap supply, the practice of disinterring bodies, and of receiving them, would of necessity be entirely abandoned.

Before adverting to those new methods for obtaining an adequate supply of subjects which have been suggested by the witnesses who have been examined before the Committee, they will state in what manner, according to the evidence adduced, the schools of anatomy at Paris are provided. They have also inquired into the practice of some other foreign schools, for an account of which they beg to refer to the evidence itself; and they dwell upon the practice of the schools of Paris, because it approaches most nearly to the plan recommended by most of the witnesses for adoption in this country.

The administration of all the hospitals at Paris, since the period of the revolution, has been confided to a public board of management. The rule at the hospitals is, that every patient who dies shall be attended by a priest, and that, after the performance of the usual ceremonies of the Catholic Church, the body shall be removed from the chapel attached to the hospital to the dead room, and there remain for twenty-four hours, if not sooner claimed by the relatives. Bodies may be examined after death, by the medical officers attached to a hospital, in order to ascertain the cause of death; but may not be dissected by them. A body, if claimed by the friends after examination, is sewed up in a clean cloth, before being delivered to them. If not claimed within twenty-four hours after death, after being enveloped in a cloth in a similar manner, it is sent, in the manner hereafter described, to one of the dissecting schools.

There are no private dissecting schools at Paris, but two public ones; that of the Ecole de la Medecine, and that adjoining the Hôpital de la Pitié. These are supplied exclusively from the different hospitals and from the institutions for maintaining paupers, the supply from certain of these establishments being appropriated to one school, and that from the remaining establishments to the other.

The distribution of subjects to the two schools is confided to a public officer, the Chêf des travaux Anatomiques. He causes them to be conveyed from the hospitals at an early hour, in a covered carriage, so constructed as not to attract notice, to a building at the schools set apart for that purpose. They are then distributed by the prosecteurs to the students; and after dissection, being again enveloped in cloth, are conveyed to the nearest place of interment.

The students at the Ecole de la Medecine consist of young men who have distinguished themselves at a public examination, though the person at the head of the establishment is also allowed to admit pupils to dissect. The school of La Pitié is open to students of all nations, who, on entering themselves, may be supplied with as many subjects as they require, at a a price varying according to the state in which the body is, from three to twelve francs; priority of choice, however, being given to the élèves internes of the different hospitals, and the subjects being delivered to them at a reduced price. English surgeons were here permitted, until lately, to engage private rooms for the purpose of lecturing on anatomy to students of their own nation, and to superintend their labours in the dissecting-room. From the protection and facilities which have thus been afforded to the study of anatomy at Paris, it has become the resort of the medical students of all nations; the practice of exhumation is wholly unknown, and the feelings of the people appear not to be violated.

It is the opinion of almost all the witnesses, that the adoption in this country of a plan similar in most respects to that which prevails in France, would afford a simple and adequate remedy for the existing evils. They recommend that the bodies of those who during life have been maintained at the public charge, and who die in work-houses, hospitals, and other charitable institutions, should, if not claimed by next of kin within a certain time after death, be given up, under proper regulations, to the anatomist; and some of the witnesses would extend the same rule to the unclaimed bodies of those who die in prisons, penitentiaries, and other places of confinement. In the hospitals which supply subjects to the anatomical schools of France and Italy, religious rites are paid to the dead before giving up the bodies for dissection: in the plan proposed for this country, most of the witnesses recommend that the performance of religious rites should be deferred until after dissection, and they are anxious that the anatomist should be required, under adequate securities, or a system of effective superintendence, to cause to be administered, at his own expense, to the bodies which he dissects, religious solemnities and the usual rites of burial.

The plan proposed has this essential circumstance to recommend it—that provided it were carried into effect, it would yield a supply of subjects that, in London at least, would be adequate to the wants of the anatomist. The number of anatomical students resorting annually to London, and the number of subjects with which they ought to be supplied, have been already stated. It appears from the returns obtained by the Committee, from 127 of the parishes situate in London, Westminster, and Southwark, or their immediate vicinity, that out of 3744 persons who died in the workhouses of these parishes, in the year 1827, 3103 were buried at the parish expense; and that of these, about 1108 were not attended to their graves by any relations. There are many parishes in and around London, from which, at the time of making this report, returns had not been delivered in; but it may be inferred from those returns which have been procured, that the supply to be obtained, from this source alone, would be many times greater than that now obtained by disinterment; that when added to the supply to be derived from those other sources which have been pointed out, it would be more than commensurate to the wants of the student, and consequently, that the plan, if adopted, as meeting the exigencies of the case, would eventually be the means of suppressing the practice of exhumation.

If it be an object deeply interesting to the feelings of the community, that the remains of friends and relations should rest undisturbed, that object can only be effected by giving up for dissection a certain portion of the whole, in order to preserve the remainder from disturbance. Exhumation is condemned as seizing its objects indiscriminately—as, in consequence, exciting apprehensions in the minds of the whole community—and as outraging in the highest degree, when discovered, the feelings of relations. If selection then be necessary, what bodies ought to be selected but the bodies of those who have either no known relations whose feelings would be outraged, or such only as, by not claiming the body, would evince indifference on the subject of dissection? It may be argued, perhaps, that the principle of selection, according to the plan proposed, is not just, as it would not affect equally all classes of the public; since the bodies to be chosen would, necessarily, be those of the poor only. To this it may be replied—1st. that even were the force of this objection, to a certain degree, admitted, yet that, to judge fairly of the plan, its inconveniences must be compared with those of the existing system; which system, according to the evidence adduced, is liable in a great measure to the same objection, since the bodies exhumated are principally those of the poor. 2dly. that the evils of this, or of any other plan to be proposed on this subject, must be judged of by the distress which it would occasion to the feelings of surviving relations, and the unfairness to one or another class of the community, by the degree of distress inflicted on one class rather than another; but where there are no relations to suffer distress, there can be no inequality of suffering, and, consequently, no unfairness shewn to one class more than another.

One or two of the witnesses, who appear to be either favourable, or not opposed to the principle of the plan, speak with doubt of its success, as though it would be found impracticable to reconcile the public to its introduction; and one in particular apprehends that religious feeling may impede its adoption. An objection founded on religious feelings does not apply to the plan in question only, but would be equally valid, generally, against all dissection whatsoever; and should lead those who urge it, consistently with their own principles, to endeavour to put down altogether the study of practical anatomy.

Though it may be true that the public are to a certain degree averse to dissection, yet it is satisfactory to find several of the witnesses adducing facts to prove that those feelings of aversion are on the decline. They state that in those parish infirmaries where the bodies of those who die are examined, as the practice has become common, it has been viewed with less jealousy: that in those hospitals where a similar rule prevails, neither patients themselves are deterred from applying for admission, nor their relatives on their behalf; that the addition of public dissecting-rooms to hospitals has not produced any diminution in the number of applications for relief within the walls of those hospitals; and that, by reasoning with the friends of those who die, and by explaining to them how important it is to the art of healing that examination should take place after death, they may usually be brought to consent to the bodies of their friends being examined. Hence it is argued, that in involving the subject of dissection in mystery, as has hitherto been the case, the public have been treated injudiciously; that with proper precautions, and the light of public discussion to guide them, they may be made to perceive the importance of the study generally, and the reasonableness of the particular measure now contemplated, and that when they come to regard it as the means of suppressing exhumation, they will receive it with favour, and finally acquiesce in it.

The legislative measure which most of the witnesses are desirous of, in order to enable them to carry the plan into effect, is the repeal of any existing law, which would subject to penalties those who might be concerned in carrying the proposed plan into execution: they wish for an enactment, permissive and not mandatory, declaring that it shall not be deemed illegal for the governors of workhouses, &c. and for anatomists, the former to dispose of, the latter to receive and to dissect the bodies of those dying in such workhouses, &c. such bodies not having been claimed within a time to be specified, by any immediate relations, and due provision being made for the invariable performance of funeral rites. Some few of the witnesses, indeed, who state that they wish for the success of the plan, contemplate any legislative interference whatever in this matter with apprehension; but they do not appear to have been aware how nearly the cases decided by the courts of law, and already adverted to, would apply to persons engaged in executing the plan in question. In those cases, the bodies for the non-burying of which the defendants were severally convicted, were those of a pauper who died in a workhouse, and of a person who had suffered death as a felon. If these cases apply, as it appears they do, to persons engaged in giving up or in receiving, for other purposes than for burial, the bodies of the inmates of workhouses or of prisons, such impediments to the success of the plan cannot be removed, as these witnesses think they might be, simply by the favourable interference of the executive government, however disposed to show indulgence to the profession; but an act of the legislature can alone provide a remedy.

Amongst the measures that have been suggested for lessening the dislike of the public to dissection, is that of repealing the clause of the act of Geo. II. which directs that the bodies of murderers shall be given up to be anatomised. It appears from the returns already laid before the house, that, as regards the direct operation of this clause, on the supply of subjects, the number which it yields to the anatomist is so small in comparison of his total wants, that the inconvenience which he would sustain from its repeal would be wholly unimportant. As to its remote operation, almost the whole of the witnesses examined before the committee, and of those whose written communications will be found in the appendix, are of opinion that the clause in question, by attaching to dissection the mark of ignominy, increases the dislike of the public to anatomy, and they therefore are desirous that the clause should be repealed.

The committee would be very unwilling to interfere with any penal enactment which might have, or seem to have, a tendency to prevent the commission of atrocious crimes; but as it may be reasonably doubted whether the dread of dissection can be reckoned amongst the obstacles to the perpetration of such crimes, and as it is manifest that the clause in question must create a strong and mischievous prejudice against the practice of anatomy, the committee think themselves justified in concluding, that more evil than good results from its continuance.

The committee consider that they would imperfectly discharge their duties if they did not state their conviction of the importance to the public interests of the subject of their inquiries. As the members of the profession are well educated, so is their ability increased to remove or alleviate human suffering. As the science of anatomy has improved, many operations formerly thought necessary have been altogether dispensed with; most of those retained have been rendered more simple, and many new ones have been performed, to the saving of the lives of patients which were formerly thought impossible. To neglect the practice of dissection would lead to the greatest aggravation of human misery; since anatomy, if not learned by that practice, must be learned by mangling the living. Though all classes are deeply interested in affording protection to the study of anatomy, yet the poor and middle classes are the most so; they will be the most benefited by promoting it, and the principal sufferers by discouraging it. The rich, when they require professional assistance, can afford to employ those who have acquired the reputation of practising successfully. It is on the poor that the inexperienced commence their practice, and it is to the poor that the practice of the lower order of practitioners is confined. It is, therefore, for the interest of the poor especially, that professional education should be rendered cheap and of easy attainment; that the lowest order of practitioners (which is the most numerous), and the students on their first entry into practice, may be found well instructed in the duties of their profession.

Such, on an attentive consideration of the evidence adduced, is the deliberate judgment of the committee on the matters submitted to them; and it now remains for the house to consider whether it will not be expedient to introduce, in the course of the ensuing session, some legislative measure which may give effect to the recommendations contained in the present report.

July 22, 1828.

### REMARKS.

In comparing the foregoing report of the committee with the immense mass of evidence from which it has been compiled, we were struck with one important defect—if not an actual dereliction of duty, on the part of the committee. Among the numerous personages which were *summoned* to afford information on the subject of inquiry—or who *volunteered* their services—there were characters from high and from low situations in life—dissection-men and resurrection-men—sergeant-surgeons to his Britannic Majesty—and (so it is said) DISSECTING-SURGEONS to his SATANNIC MAJESTY. Be the latter true or false, there presented himself before the committee a dissector of great experience, whose evidence occupies some quarto pages of the original minutes. This deponent threw a *new light* upon the grave subject—and traced, with great ingenuity and truth, the evil to its primary source. He proved to a demonstration, that the difficulties attendant on the prosecution of anatomy —in other words, the *scarcity of subjects*, was solely owing to the CHARTER OF THE COLLEGE OF SURGEONS! The cause being discovered, the remedy became obvious. It was "CITO TUTÉ, AC JUCUNDÉ." The Parliament had only to blow up the Temple of Esculapius in Lincolns-Inn-Fields, with all the "BATS AND CORRUPTIONISTS," thereunto belonging, and every anatomist in town and country would instantly be surrounded by shoals of dead bodies, as was the heroic Grecian by shoals of ghosts, when he descended into the regions of Tartarus. The dissector-general not only exposed the evil and proposed the remedy; but he volunteered to *execute* the latter—viz. to *set fire* to the faggots, and make an "AUTO DA FÉ" of the whole council, not even excepting Mr. LAWRENCE!*

Now considering that the subject of inquiry was EXHUMATION—that the above important information was tendered by a no less respectable personage than the dissector-general of his Subterranean Majesty—that the evil was rendered clear, and the remedy easy—then we say that the committee has insulted the dead, and injured the living, by passing over, in total silence, the valuable suggestions and disinterested offers of the DISSECTOR aforesaid. Not a single allusion is made in the report to a body of parole evidence occupying whole quarto pages of the minutes! Can the committee have leagued themselves with the "Bats and Corruptionists" of the College? This cannot be; since the Dissector lauded them to the skies during the time they were sitting. In short, this conduct of the committee gives us reason to fear that there is a general conspiracy among all the upper classes of society, professional and non-professional, to drown the voice of truth, honour, and impartiality in the personage whose cause we are here advocating.

---

### 73. COLLEGE OF SURGEONS—MR. LAWRENCE.

We have little leisure, and less inclination for MEDICAL POLITICS—especially where the concerns are more of a personal than a public nature. The election of Mr. Lawrence, however, into the council of the College of Surgeons, and his acceptance of office, are events which could have been little anticipated two years ago, and which are calculated to excite various reflexions in the most

* We are happy to see that Mr. L. has become a BENEDICT as well as a COLLEGE COUNCILMAN. He will now be useful to the State in various ways, and we wish him success in both capacities.

astute minds. We should not hold ourselves exculpable if we indulged in retrospective observations calculated to harrow up any unpleasant reminiscences in the breasts of Mr. Lawrence or of the members of the College council. No man who has the interest of his profession, or the dignity of its members at heart, would wish to do so at the present time. When a man changes his party, his tenets, or his professions, he lays himself open to the charge of inconsistency—and sometimes to more serious insinuations; but, for our own parts, we see no just cause for censure in such cases, unless the *motives* which prompt to the change of sentiment, be proved to be CORRUPT. MUTABILITY is one of the most distinguishing characters of the human mind, and where is the man who has resolutely clung to the same opinions through life?

Manners with fortunes, tempers turn with climes,
Tenets with books, and principles with times.

Is a man culpable for relinquishing an error which he had imbibed—-for embracing a tenet which he had doubted—for withdrawing from a party which he did not approve? By no means. Finally, is it culpable or laudable to forget and forgive animosities—to sail down the stream of life (rough enough of itself) at peace with our fellow-travellers—to foster the kinder passions of our nature, and repress the malevolent—to cherish unanimity in a liberal profession, and discountenance the promoters of feuds and dissentions?* These questions can only be answered in one way. We conceive that the College shewed its wisdom as well as its liberality in the election of Mr. Lawrence—and we think that Mr. Lawrence acts an honorable part in sacrificing all personal considerations for the sake of healing the wounds under which the profession has long smarted. We have good reasons to know that Mr. Lawrence has been the unwilling—perhaps even the unconscious instrument by which much mischief has been perpetrated during the last two years. His *temporary* association with a man, whose writings have sunk the medical character of this country in the eyes of all Europe, before the mask was torn away, has been dexterously converted into an implied alliance with the arch calumniator ever since! Hence the satirical abuse of a *fiery* demagogue was too generally considered to be approved by a man whose professional talent and moral character lent to that abuse the only passport which could have circulated it among respectable society. All this was a dark imposition, a cunning device of the enemy of harmony in the profession. No event could more effectually dispel the last fleeting clouds of that moral tempest which has so long been carrying on its work of devastation, than this junction of Mr. Lawrence with the councils of the College. There will be no pretext now for associating his name with the defamatory harrangues of the "*moral* incendiary." The Freemason's Tavern will no more ring the tocsin of medical revolution. The torch of discord is burnt to the very end—and the lurid glimmerings which it emits, serve only to shadow forth the bloody arm of a public executioner:—we do not mean the executioner at Newgate, who strangles the victim of offended justice—but the LITERARY ASSASSIN who stabs the character of all indiscriminately who come in his way, and who happen to be a degree elevated above himself!* It is to be hoped that now the liberal part of the council has got the ascendancy, they will endeavour to promote the dignity and utility of the surgical profession, by the promulgation of wise laws and salutary regulations, so as to deprive the enemies of order of all handle for vituperation and abuse. We are not so unjust as to attribute all the

* We have the very best means of knowing that no man was ever more sick or ashamed of the company into which he inadvertently dropped, than Mr. Lawrence—and that no man is more anxious to put an end to the disgraceful scenes of vituperation which have so long been held up to the disgusted view of every respectable member of the profession.

* It looks somewhat strange that two numbers of the Lancet should be published, *after Mr. Lawrence's election*, without the slightest notice being taken of that event. Previously to this epoch, Mr. Lawrence could scarcely sneeze without its being gazetted in the Lancet! How is this silence to be accounted for?

evils that exist in medical society to the mal-administration of the three corporate bodies—nor so Utopean as to expect that their edicts alone can purify the profession. The evils lie deep in the human heart—and few of them can be reached by human laws! Envy, hatred, uncharitableness, have been too long the order of the day, and these do more to injure the members of our Profession in the eyes of the public at large, than fifty colleges or corporations, however viciously constructed.*

---

74. FORMATION OF AN HERBARIUM.

We recommend the specimens to be dried between leaves of paper, under the pressure of a bag of sand or small shot, and, when perfectly dry, transferred to a bound specimen-book, and sewed (not gummed) to its leaves, in the order of gathering the specimens. The name should be attached to each specimen by a slip of paper. In the course of three or four years, two or three thousand specimens will have been dried, and these may then be transferred to another book or books, arranged according to the natural system, gummed on, and their names, &c. written beside them. The mode of forming the book for this purpose is as follows. —Suppose the size to be folio; then gum the specimens on one side of leaves of drawing cartridge-paper, paste a leaf of reddish-brown blotting-paper on the back of the leaf containing the specimens, and paste a margin of cartridge-paper all round both sides of each leaf, say half an inch broad at top and bottom, and the outer edge, and one inch broad at the inner or binding edge. This being done, put each leaf separately in a press, and let it remain there till it is pressed quite flat. Proceed in the same way with as many separate leaves as will contain all the specimens to be arranged, or as many as will make a proper sized volume; and then send them to a binder, to be bound in the usual way. The effect of the double edge will be, that each page of specimens will, as it were, lie fastened to the bottom of a shallow drawer, completely excluded from the air; and the volume may be kept in a book-case along with others, in the usual way, and, by its index, referred to with as much ease as any other botanical work. This is by far the best method of keeping specimens that we know of, for small collections; and we would strongly recommend all those who can afford it, to employ Professor La Gasca (*Gard. Mag.* Vol. II. p. 220) to form books of this sort, containing one or two species under each order and tribe of the natural system. There could not be purchased a work of equal value to the young botanist. Mr. Toward, flower-gardener to the Duchess of Gloucester, is the only person that we know of who possesses an herbarium done up in this way, and to him belongs the merit of the invention. The binder was Mr. Perryman, of Windsor, himself much attached to botany. (*Gard. Mag.* August.)

* It was but the other day that a young man was prosecuted at Bristol, in consequence of phlebitis succeeding venesection. It was sworn in evidence that, the first remark he made, on baring the patient's arm, conveyed a direct reflexion on the *unskilfulness* of those who had bled the patient previously! This accusation too was pronounced merely from looking at the scars! The accident which followed the venesection appears almost as a judgment on the illiberal and unprofessional conduct of the youngster. We say unprofessional;—the proper word would perhaps be—*professional!* The rising generation are systematically taught to traduce their superiors—and the more falshoods they use, the greater is the indication of their talent. This recent substitution of defamation for ethics, is a source of much greater calamity to the profession than all the abuses of all the institutions in the empire. The LATTER may occasion injustice in the case of a few individuals—the FORMER strikes at the very fountain of honour, honesty, and virtue, by corrupting the heart and rending asunder every social tie by which medical society ought to be held together. It is a pity that a Court of Honour is not established in the medical profession, to which its members might appeal in cases that are incognizable by the laws. There is no profession in which so much mischief may be done, by means which the law cannot possibly notice, as in the medical.
